# Genetic Diversity of MHC B-F/B-L Region in 21 Chicken Populations

**DOI:** 10.3389/fgene.2021.710770

**Published:** 2021-08-13

**Authors:** Yiming Yuan, Huanmin Zhang, Guoqiang Yi, Zhen You, Chunfang Zhao, Haixu Yuan, Kejun Wang, Junying Li, Ning Yang, Ling Lian

**Affiliations:** ^1^National Engineering Laboratory for Animal Breeding and MOA Key Laboratory of Animal Genetics and Breeding, College of Animal Science and Technology, China Agricultural University, Beijing, China; ^2^United States Department of Agriculture, Agricultural Research Service, Avian Disease and Oncology Laboratory, East Lansing, MI, United States; ^3^Shenzhen Branch, Guangdong Laboratory of Lingnan Modern Agriculture, Genome Analysis Laboratory of the Ministry of Agriculture and Rural Affairs, Agricultural Genomics Institute at Shenzhen, Chinese Academy of Agricultural Sciences, Shenzhen, China; ^4^College of Animal Science and Technology, Henan Agricultural University, Zhengzhou, China

**Keywords:** chicken, MHC, target enrichment sequencing, B-F/B-L region, genetic diversity

## Abstract

The chicken major histocompatibility complex (MHC) on chromosome 16 is the most polymorphic region across the whole genome, and also an ideal model for genetic diversity investigation. The MHC B-F/B-L region is 92 kb in length with high GC content consisting of 18 genes and one pseudogene (Blec4), which plays important roles in immune response. To evaluate polymorphism of the Chinese indigenous chickens as well as to analyze the effect of selection to genetic diversity, we used WaferGen platform to identify sequence variants of the B-F/B-L region in 21 chicken populations, including the Red Jungle Fowl (RJF), Cornish (CS), White Leghorns (WLs), 16 Chinese domestic breeds, and two well-known inbred lines 6_3_ and 7_2_. A total of 3,319 single nucleotide polymorphism (SNPs) and 181 INDELs in the B-F/B-L region were identified among 21 populations, of which 2,057 SNPs (62%) and 159 INDELs (88%) were novel. Most of the variants were within the intron and the flanking regions. The average variation density was 36 SNPs and 2 INDELs per kb, indicating dramatical high diversity of this region. Furthermore, *BF2* was identified as the hypervariable genes with 67 SNPs per kb. Chinese domestic populations showed higher diversity than the WLs and CS. The indigenous breeds, Nandan Yao (NY), Xishuangbanna Game (XG), Gushi (GS), and Xiayan (XY) chickens, were the top four with the highest density of SNPs and INDELs. The highly inbred lines 6_3_ and 7_2_ have the lowest diversity, which might be resulted from a long-term intense selection for decades. Collectively, we refined the genetic map of chicken MHC B-F/B-L region, and illustrated genetic diversity of 21 chicken populations. Abundant genetic variants were identified, which not only strikingly expanded the current Ensembl SNP database, but also provided comprehensive data for researchers to further investigate association between variants in MHC and immune traits.

## Introduction

China has the most abundant resources of domestic chickens, which possess highly genetic polymorphisms. As a highly polymorphic region across the genome, major histocompatibility complex (MHC) is a group of tightly linked genes, encoding major histocompatibility antigens, which discriminate between self and non-self cells and regulate immune responses. MHC is closely related to disease resistance and production performance of animals (Bacon, 1987). Chicken MHC-B and MHC-Y regions reside on the micro-chromosome 16, which are located on the same side of nucleolar organizing region (NOR) (Miller and Taylor, 2016). MHC-B and MHC-Y are inherited independently because they are separated by a GC rich region (Miller et al., 2004). Until now, 46 genes within a 2,41,833 bp in MHC-B were identified (Shiina et al., 2007), including three highly polymorphic gene groups: *BF*, *BL*, and *BG*, which encode class I, class II, and class IV glycoproteins on cell surface, respectively (Lamont et al., 1987). The encoded proteins play important roles in rapid allograft rejection, immune response and determining susceptibility/resistance to pathogen infections (Pazderka et al., 1975; Plachy et al., 1992; Kaufman, 2018).

The BF/BL region (Guillemot et al., 1988), a 92-kb core region with a relatively high GC content (Shiina et al., 2007), has been described as a minimal essential MHC region since it contains two classical class I (B-F), two classical class II B (B-LB) genes, and some other genes involved in antigen processing and presentation (Bacon, 1987). This region contains 18 genes and 1 pseudogene (*Blec4*) (Lamont et al., 1987; Kaufman et al., 1995), of which, two MHC class I genes *BF1* and *BF2* encode α chain of BF antigen (Ewald and Livant, 2004). *BLB1* and *BLB2* encode β chains of classic MHC class II molecules (Lamont, 1989; Zekarias et al., 2002). MHC is a polymorphic region that has numerous SNPs and INDELs (Wong et al., 2004), and its variations can represent the whole genome-level variation to evaluate genomic diversity and population history (Yuhki and Obrien, 1990). The microsatellite locus *LEI0258*, a tetranucleotide repeat system located within the BF/BL region, has been used to analyze population genetic diversity, structure, distinctiveness, and relationships between populations (McConnell et al., 1999; Fulton et al., 2006; Izadi et al., 2011; Chang et al., 2012). Huang et al. (2016) used *LEI0258* to examine the genetic diversity and evolutionary history of south China domestic chickens and revealed that south China domestic chickens mainly originated from the Red Jungle Fowl (RJF). Han et al. (2013) found high genetic diversity of the MHC region in Chinese indigenous chickens by assessing genetic information on *LEI0258*. In addition to LEI0258, there are also many microsatellite loci such as MCW0370 and MCW0371. The use of these high polymorphism markers is of great significance for the study of chicken MHC diversity (Esmailnejad et al., 2017; Manjula et al., 2021).

However, to the best of our knowledge, there is no single-base-resolution polymorphism description of MHC BF/BL region in Chinese domestic chickens. In this study, we comprehensively evaluated the polymorphisms of BF/BL region among 16 Chinese domestic populations, as well as White Leghorns (WLs), Cornish (CS), RJFs, and two high inbred lines (lines 6_3_ and 7_2_) (Waters, 1945; Stone, 1975; Bacon et al., 2000) using the WaferGen platform, which is a more cost-effective method for identifying variations in targeted regions (De Wilde et al., 2014).

## Materials and Methods

### Ethics Statement

All experimental procedures and used animals were approved by the Animal Care and Use Committee of China Agricultural University.

### Sample Selection

A total of 195 chickens from 21 populations were used in this study. Sixteen were Chinese domestic chicken populations (Table 1), including Dagu (DG, *n* = 9), Wenchang (WC, *n* = 10), Wuding (WD, *n* = 10), Xiayan (XY, *n* = 10), Xishuangbanna Game (XG, *n* = 10), Luxi Game (LX, *n* = 7), Gushi (GS, *n* = 10), Wenshang Barred chicken (WS, *n* = 10), Nandan Yao (NY, *n* = 10), Yunyang Black-bone chicken (YY, *n* = 10), Tibetan (TB, *n* = 10), Beijing You (BY, *n* = 10), Chahua (CH, *n* = 10), Dongxiang Blue-eggshell chicken (DX, *n* = 10), Shouguang (SG, *n* = 10), and Silkie (SK, *n* = 10). Two introduced populations were CS(*n* = 10) and WL(*n* = 10). The blood samples of first ten local breeds were collected previously by our colleagues from local chicken breed preservation farm. The blood samples of latter six local breeds as well as CS and WL were from Experimental Farm of Poultry Resource in China Agricultural University. The individuals were randomly selected for the assay. RJF (*n* = 3) was also included in this study, and DNA was kindly provided by Dr. Xiaoxiang Hu. Two highly inbred WL lines 6_3_ (*n* = 8) and 7_2_ (*n* = 8) were that were cultivated by the Avian Disease and Oncology Laboratory (ADOL) of the United States Department of Agriculture. After decades of breeding, the inbreeding coefficient of the two lines reached 99%. Serological tests showed that the two lines have the same MHC *B* haplotype (*B2*). Although they have the same haplotype, there were significant differences in Marek’s disease (MD) resistance: line 6_3_ is resistant to MD tumors but susceptible to both Marek’s disease virus (MDV) and avian leukosis virus (ALV). Conversely, the line 7_2_ is resistant to ALV infection but susceptible to both MDV and MD tumors (Waters, 1945; Stone, 1975; Bacon et al., 2000). DNA of line 7_2_ and 6_3_ were extracted from spleens.

**TABLE 1:** The distribution of domestic chicken breeds in China.

Breeds	Origins
Beijing You	Beijing
Chahua	Yunnan
Dagu	Liaoning
Dongxiang Blue-eggshell	Jiangxi
Gushi	Henan
Luxi Game	Shandong
Nandan Yao	Guangxi
Shouguang	Shandong
Silkie	Jiangxi
Tibetan	Tibet
Wenchang	Hainan
Wuding	Guangxi
Wenshang Barred	Shandong
Xishuangbanna Game	Yunnan
Xiayan	Guangxi
Yunyang Black-bone	Hubei

### Target Enrichment Sequencing

Genome DNA were extracted from blood or tissue samples using DNA extraction kit (TIANGEN). The purity, concentration, and volume of the extracted DNA were tested. WaferGen Smartchip platform was used to perform highly parallelized PCR for the target region and the procedure was followed that by De Wilde et al. (2014). There were total of 431 amplicons were designed using tiling settings, taking into account known SNP positions and with a target annealing temperature of 60°C to cover target region (approximately 92 kb B-F/B-L region on chromosome 16). Primer3 (Version 2.3.7) global settings for primer design, length setting of amplicons were as follows: 250–300 bp and 301–350 bp, primer length: 18–30 nt, primer pair_max_diff_Tm: 3°C, and primer TM: 58–62°C (60°C as optimal). Primers were listed in Supplementary Table 1. Specific barcodes were added to each sample. Parallelized PCR were cycled in the SmartChip Cycler (WaferGen Biosystems). Library was constructed by PCR pool. The concentration of PCR pool was measured using the dsDNA assay kit on the Qubit fluorometer (Invitrogen) and fragment analysis occurred on BioAnalyzer 2100 (Agilent). The library was sequenced by MiSeq platform.

### Reads Mapping and Variant Calling

Chicken reference genome assembly (Galgal6 version) was download from NCBI.^1^ Trimmomatic (Trimmomatic 0.39) was used to obtain clean reads, and the parameter of quality control was as follows: SLIDINGWINDOW:5:20 LEADING:3 TRAILING:3 MINLEN:36 (Bolger et al., 2014). SAMtools (Samtools 1.9) was used to create an index of the reference sequence (Li et al., 2009). Burrows–Wheeler Alignment tool (BWA-0.7.17) was used to mapping reads to the reference genome with the command “mem -t 10 -M” (Li and Durbin, 2010). The alignment data stream was piped to SAMtools and converted to a BAM file, which was then sorted according to the reference genome. The MarkDuplicates module in Genome Analysis Toolkit (GATK-4.1.3.0) was used to remove duplicated reads (McKenna et al., 2010). GATK HaplotypeCaller module and GATK GenotypeGVCFs module were used to convert BAM files to GVCF files and then switched to VCF files. The GATK VariantFiltration module was used to hard-filter SNP and INDEL. For SNP, we excluded potential false variants following these criteria: QUAL < 30.0 ∣∣ QD < 2.0 ∣∣ FS > 60.0 ∣∣ MQ < 40.0 ∣∣ SOR > 3.0 ∣∣ MQRankSum < −12.5 ∣∣ ReadPosRankSum < −8.0. For INDEL, we took out variants with the following parameters: QUAL < 30.0 ∣∣ QD < 2.0 ∣∣ FS > 200.0 ∣∣ SOR > 10.0 ∣∣ MQRankSum < −12.5 ∣∣ ReadPosRankSum < −20.0. The variants with multiple alleles were also excluded. SNPs was identified using the criteria of filtering minor allele frequency (MAF)<0.01 and missing genotype>10% in the 21 chicken populations. Subsequently, the Variants Effect Predictor (VEP) on Ensembl was used to annotate variants.

### Comparison Between Chickens Showing MD Resistance and Susceptibility

Comparisons were conducted between the line 6_3_ and line 7_2_. Allele frequency and frequency difference of each SNP were calculated with VCFtools (versions 4.0) – freq and –diff-site (Danecek et al., 2011). In each of the lines, loci with genotypic information was retained from more than four individuals. The difference of allele frequency among two populations was subjected to chi-squared testing.

## Results

### Sequencing and Mapping Summary

**FIGURE 1:**
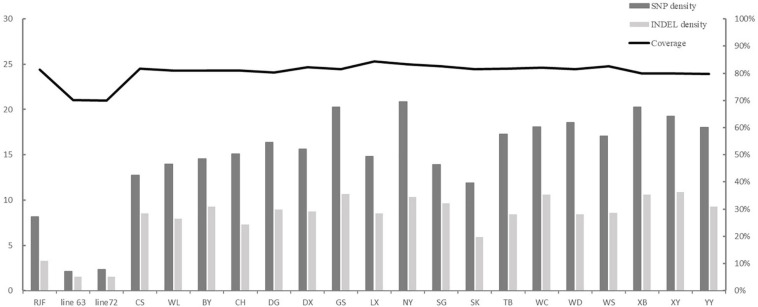
SNP and INDEL density in each population. The variation density means that the count of SNPs per kb and the count of INDELs per 10 kb.

On average, ∼1,14,000 (80%) clean reads were obtained from ∼1,42,000 raw reads for each individual. Eighty-eight percentage of reads were aligned to the chicken genome assembly (galGal6) (Table 2), and the captured specificity reached 99%. The coverage of target regions ranged from 70 to 84%, with an average of 80%. However, the target region coverage of the line 6_3_ and line 7_2_ was the lowest, about 70% (Figure 1). The average depth of the 92 kb target regions reached over 240×, among them, the coverage of 55 kb was more than 30×, and the coverage of 64 kb was more than 10×.

**TABLE 2:** Summary statistics of a targeted sequencing and mapping region of chicken major histocompatibility complex (MHC).

Population name^a^	Clean reads	Mapping rate^b^ (%)	Capture specificity^c^ (%)	Mean depth of target region (X)
Line 6_3_	1,14,206	86.85	99.21	234.4
Line 7_2_	1,14,488	86.56	99.18	232.9
BY	1,14,629	86.87	99.23	243.3
CH	1,17,262	89.02	99.39	255.0
CS	1,15,211	87.23	99.24	244.2
DG	1,15,510	91.05	99.39	255.8
DX	1,14,403	88.78	99.28	245.1
GS	1,10,158	88.79	99.34	238.3
LX	1,14,702	90.94	99.43	252.1
NY	1,13,370	90.11	99.23	246.4
RJF	1,12,549	83.34	99.24	224.3
SG	1,15,861	89.56	99.24	252.3
SK	1,13,112	86.76	99.18	238.8
TB	1,15,405	87.42	99.30	247.7
WC	1,15,773	85.65	99.14	239.5
WD	1,15,933	87.71	99.32	248.1
WL	1,15,457	88.30	99.10	248.1
WS	1,12,316	87.30	98.94	237.9
XB	1,15,583	89.19	99.37	249.6
XY	1,15,110	91.97	99.41	259.0
YY	1,02,682	81.04	98.96	204.4
Average	1,13,987	87.83	99.24	242.7

*^a^Chicken: BY, Beijing You; DG, Dagu; WC, Wenchang; WD, Wuding; XY, Xiayan; XB, Xishuangbanna Game; TB, Tibetan; CH, Chahua; LX, Luxi Game; GS, Gushi, WS, Wenshang Barred chicken; DX, Dongxiang Blue-eggshell; NY, Nandan Yao; SG, Shouguang; SK, Silkie; YY, Yunyang Black-bone; CS, Cornish; WL, White Leghorn. Line63, Marek’s disease resistant populations; Line72, Marek’s disease susceptible populations; RJF, Red Jungle Fowl.*

*^b^The reads aligned to genome accounted for the clean reads.*

*^c^The reads aligned to target region accounted for the reads aligned to genome.*

The SNP database at Ensembl (release 102) had 4,804 known variations in this region, including 4,572 SNPs and 232 INDELs. In this study, a total of 3,319 SNPs and 181 INDELs were identified in the 21 populations (Table 3). Among them, there were 1,262 known SNPs (38%) and 22 known INDELs (12%); and the rest 2,057 SNPs (62%) and 159 INDELs (88%) were discovered for the first time. The Chinese domestic populations GS, NY, XB, and XY had the most abundant SNPs and INDELs than others, and the highly inbred line 6_3_ and 7_2_ showed the fewest SNPs and INDELs among the populations. There were no breed-specific SNPs or INDELs identified in this study, and most of them were mutually shared between some populations. We also calculated the allele frequency to analyze the difference in the BF/BL region between the MD resistant line 6_3_ and the MD-susceptible line 7_2_. There were only 12 loci with allele frequency differences greater than 0.3, and the largest difference was 0.5, seven of them differed at significant levels (*P* < 0.05, chi-squared testing). There was no SNP locus that completely separates the two lines (Table 4).

**TABLE 3:** SNPs and INDELs detected in 21 chicken populations.

Breed	SNP count		INDEL count				Maximum length(bp)	
	Total	Novel (ratio)	Total	Insertion	Deletion	Novel (ratio)	Insertion	Deletion
RJF	618	249(40.3%)	25	7	18	19(76.0%)	7	31
Inbred population	254	175(68.9%)	14	2	12	12(85.7%)	2	60
Line 6_3_	139	85(61.2%)	10	0	10	9(90.0%)	0	60
Line 7_2_	153	98(64.1%)	10	2	8	9(90.0%)	2	60
Introduced breed	1,538	671(43.6%)	92	25	67	74(80.4%)	15	60
CS	971	430(44.3%)	65	20	45	51(78.5%)	11	60
WL	1,056	364(34.5%)	60	16	44	48(80.0%)	15	60
Domestic breed	2,862	1,671(58.4%)	157	42	115	137(87.3%)	25	60
BY	1,098	480(43.7%)	70	20	50	57(81.4%)	8	60
CH	1,138	487(42.8%)	55	13	42	43(78.2%)	11	60
DG	1,224	499(40.8%)	67	20	47	59(88.1%)	15	44
DX	1,197	484(40.4%)	67	22	45	55(82.1%)	25	60
GS	1,536	704(45.8%)	81	25	56	69(85.2%)	15	60
LX	1,163	486(41.8%)	67	17	50	53(79.1%)	7	60
NY	1,619	724(44.7%)	80	23	57	64(80.0%)	25	60
SG	1,072	458(42.7%)	74	24	50	64(86.5%)	8	44
SK	902	379(42.0%)	45	13	32	36(80.0%)	7	60
TB	1,314	555(42.2%)	64	23	41	50(78.1%)	15	34
WC	1,382	592(42.8%)	81	25	56	65(80.2%)	15	60
WD	1,409	595(42.2%)	64	18	46	54(84.4%)	11	60
WS	1,311	541(41.3%)	66	16	50	50(75.8%)	11	60
XB	1,510	658(43.6%)	79	22	57	65(82.3%)	15	60
XY	1,435	643(44.8%)	81	24	57	66(81.5%)	15	44
YY	1,340	585(43.7%)	69	19	50	52(75.4%)	11	44

**TABLE 4:** Allele frequency differences between the line 6_3_ and line 7_2_.

Position	Gene	Line 63			Line 72			Difference	*P*-value
		Allele count	Ref	Alt	Allele count	Ref	Alt		
2533677	BTN1	10	G:0.8	A:0.2	8	G:0.5	A:0.5	0.3	0.1596
2537585	BTN1	12	C:1	T:0	10	C:0.6	T:0.4	0.4	0.0154
2547609	BTN2	8	A:0.5	G:0.5	10	A:1	G:0	0.5	0.0112
2547628	BTN2	8	T:0.5	C:0.5	10	T:1	C:0	0.5	0.0112
2547651	BTN2	8	T:0.5	G:0.5	10	T:1	G:0	0.5	0.0112
2586535	DMA	12	G:0.33	A:0.67	8	G:0	A:1	0.33	0.0679
2591649	DMB2	8	A:0.25	G:0.75	10	A:0.6	G:0.4	0.35	0.1376
2593501	BF1	8	A:0.5	AGC:0.5	10	A:0.2	AGC:0.8	0.3	0.1797
2597541	TAP1	10	T:0.3	A:0.7	12	T:0	A:1	0.3	0.0412
2599123	TAP1	10	T:0	G:1	12	T:0.33	G:0.67	0.33	0.0435
2604977	Downstream of TAP2	8	G:0	T:1	10	G:0.3	T:0.7	0.3	0.0897
2610020	Upstream of C4	10	G:0.6	A:0.4	12	G:1	A:0	0.4	0.0154

Since the coverage of the different populations was different, we calculated variation density as follows: the numbers of SNPs and INDELs were divided by the coverage of each population. The population diversity was assessed as the number of SNP per kb and the number of INDEL per 10 kb. The top five populations with the highest density of SNPs were NY, XB, GS, XY, and WD, with 20.87, 20.29, 20.26, 19.28, and 18.57 per kb, respectively. The lowest density of SNP was found in RJF, inbred line 6_3_ and line 7_2_, with 2.13, 2.35, and 8.16, respectively. Similarly, NY, GS, XB, XY, and WC five populations showed the highest density of INDELs, and the three populations with the lowest density of INDELs were RJF, the line 6_3_, and line 7_2_. The SNP and INDEL densities in the two introduced populations, CS and WL, were lower than those of most of the Chinese indigenous populations (Figure 1).

### Characteristic and Distribution of SNPs in Genes

**FIGURE 2:**
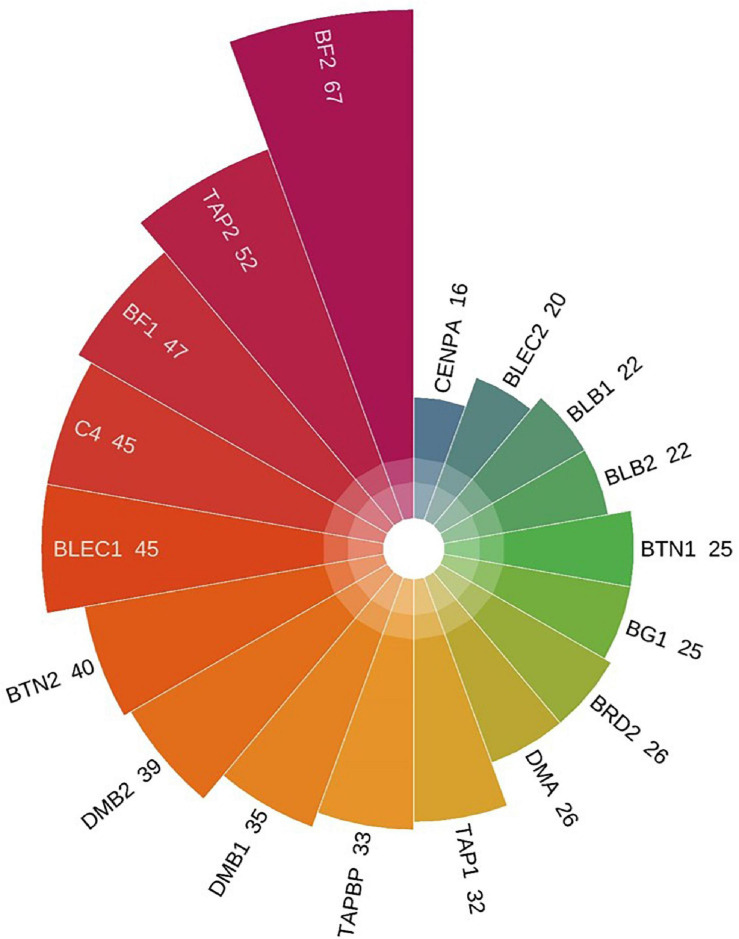
The number of SNPs per kb in 18 genes. The SNP rates from low to high follow the clockwise direction.

**FIGURE 3:**
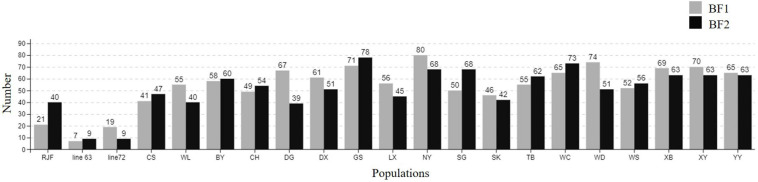
The number of SNPs in *BF1* and *BF2* among different chicken populations.

**FIGURE 4:**
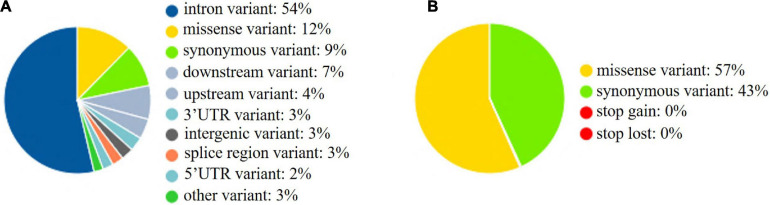
SNPs in functional regions. **(A)** SNPs in functional regions. **(B)** SNPs in coding sequences. upstream: 500 bp apart from the transcription start site; downstream: 500 bp apart from the transcription end site.

The whole BF/BL region showed a very high genetic diversity, with an average of about 36 SNPs per kb. Among a total of 18 genes, the most diverse genes were *BF2* and *TAP2*, with more than 50 SNPs per kb. About 86% of the SNPs identified in *BF2* were newly discovered. The coding region variation was primarily concentrated in the second and third exon of *BF2. BF1* and *C4* followed with more than 40 SNPs per kb. *CENPA*, *BLEC2*, and two *BLB* genes had the lowest SNP rates of about 20 SNPs per kb (Figure 2). Among the 21 populations, RJF, line 6_3_ and line 7_2_ had the lowest diversity, while NY, GS, and XG were observed with the highest diversity in both BF genes (Figure 3). The diversity of CS and WL were lower than most of the Chinese domestic breeds. In addition, we also counted the number of variants in other genes (Supplementary Table 2). We then annotated all detected SNPs using VEP and found that most SNPs (54%) were located on the intron, 21% in coding region, about 7 and 4% in the downstream and upstream flanking regions, respectively, 3% in splice region and 5% were situated at UTR regions (Figure 4A). In the coding region, more than half of the SNP were missense variants; 43% were synonymous variants (Figure 4B).

### Characteristic and Distribution of INDELs in Genes

**FIGURE 5:**
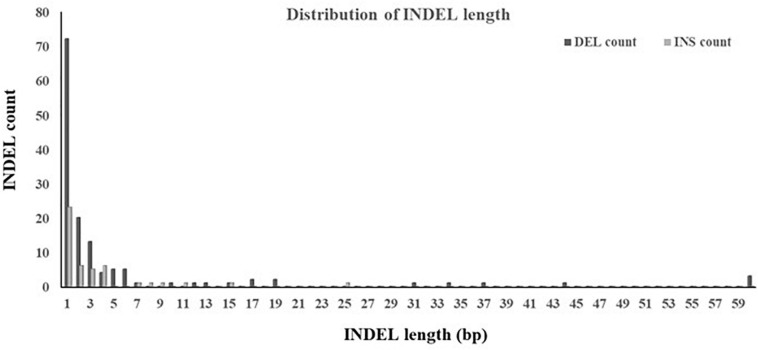
Distribution of INDEL length. INDELs with multiple alleles were not included.

The ratio of deletion (64%) was significantly higher than that of insertion (36%). Both the largest deletion and insertion detected in this study were 60 and 25 bp, respectively, and both the smallest deletion and insertion were 1 bp. A large proportion (89%) of INDELs were less than 10 bp. The majority was the single base-pair INDEL and accounted for 58% of all detected INDELs. In addition, INDELs were spread out of a 826 bp segment, accounting for 1% of the target region, and the region affected by deletions was larger than that by insertions (Figure 5).

**FIGURE 6:**
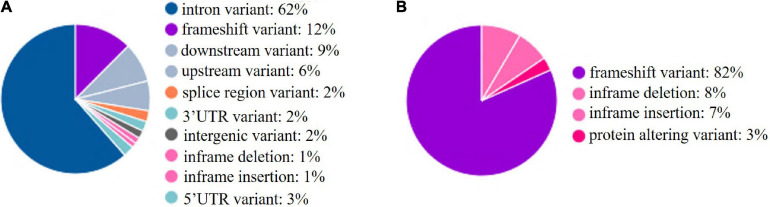
INDELs in functional regions. **(A)** INDELs in functional regions. **(B)** INDELs in coding sequences. upstream: 500 bp apart from the transcription start site; downstream: 500 bp apart from the transcription end site.

To explore the distribution of INDELs across the 18 genes in the BF/BL region, insertion and deletion of different genes were counted, and the INDEL rate was calculated as the number of INDEL per kb for each gene. We observed that the average of INDEL rate on the target region was 2 per kb. The *BLEC2* and *TAP2* showed the lowest INDEL rate. The two genes, *BF1* and *BF2*, coding the components of MHC class I molecules, showed the highest INDEL rate. Both showed more than 5 INDELs per kb. Our data showed that the *BF1* and *BF2* did not have large insertions and deletions, and the length of the INDELs in these genes was within 15 bp (Table 5). In order to have a deeper insight on the distribution of INDELs in the gene regions, we annotated all the detected INDELs using VEP. We found that 62% INDELs were located on introns, 14% in coding exon, 15% in downstream and upstream flanking region, 5% in the UTR regions, and 2% in splice region (Figure 6A). In the coding region, more than three-quarters of the identified variants were frameshift variant, 8% were inframe deletion variant, 7% were inframe insertion variant, and the remaining 3% were protein altering variant (Figure 6B).

**TABLE 5:** Distribution of INDELs across 18 genes.

Gene	Length	INDEL count			Maximum length (bp)		INDEL rate(kb^–^^1^)	Novel (ratio)
		Num	Insertion	Deletion	Insertion	Deletion		
BTN1	14,220	28	8	20	7	60	1.9691	22(78.6%)
BTN2	6,884	14	2	12	2	60	2.0337	12(85.7%)
BG1	5,195	10	2	8	11	3	1.9249	9(90.0%)
BLEC2	2,493	1	0	1	0	3	0.4011	1(100%)
BLEC1	4,150	9	3	6	15	6	2.1687	8(88.9%)
BLB1	1,868	2	2	0	4	0	1.0707	2(100%)
TAPBP	3,532	7	2	5	2	5	1.9819	6(85.7%)
BLB2	1,537	1	0	1	0	1	0.6506	1(100%)
BRD2	8,854	8	3	5	1	6	0.9035	6(75.0%)
DMA	2,210	2	0	2	0	60	0.9050	2(100%)
DMB1	2,462	4	1	3	1	37	1.6247	4(100%)
DMB2	3,024	12	2	10	25	15	3.9683	9(75.0%)
BF1	2,803	15	4	11	4	12	5.3514	15(100%)
TAP1	5,195	4	1	3	4	2	0.7700	4(100%)
TAP2	3,563	2	0	2	0	2	0.5613	1(50.0%)
BF2	2,204	13	6	7	4	3	5.8984	13(100%)
C4	14,355	27	3	24	3	60	1.8809	24(88.9%)
CENPA	1,610	4	2	2	1	2	2.4845	4(100%)

## Discussion

Next generation sequencing (NGS) has been widely used for identification of genome-wide genetic variation (Davey et al., 2011; Nielsen et al., 2011). However, chromosome 16 has not been elucidated well by NGS analysis because the sequencing coverage of chr16 was relatively poor (only about 50%), much lower than that of the other chromosomes (Yan et al., 2014). Additionally, NGS is not economically suitable for large-scale samples due to the high cost, therefore many cost-efficient methods have been developed to analyze subsets of genome-scale data, such as target enrichment sequencing of specific genes, which can significantly reduce the costs (Dapprich et al., 2016). WaferGen, a target region sequencing platform, is a PCR based platform that can generate as many as 5,184 amplification reactions on one chip (De Wilde et al., 2014). In this study, we conducted target enrichment sequencing to evaluate the polymorphism in the BF/BL region of 21 diverse chicken populations. Our capture efficiency was 99%, which was higher than a previous study in turkey (capture efficiency 53%) using a custom MHC SureSelect capture array (Reed et al., 2016). The average depth of our data in the target region reached about 200×, and the coverage of target region with more than 30× were 55 kb on average. The mean depth of the target region in this study was three times deeper than similar reported research in human (Jones and Good, 2016), which indicated high accuracy and reliability of the variations identified in this study.

A total of 3,500 variations were detected in the BF/BL region of the 21 chicken populations in this study. Among them, 2,057 SNPs and 159 INDELs were novel, which drastically expand the current Ensembl SNP database. The number of novel INDELs detected in this study was quite large, accounted for 5% of the total variations, but most of them privately appeared in some populations, which suggested that using additional populations with large genetic background differences may detect more genetic variation of the genome. We identified an average of 36 SNPs and 2 INDELs per kb in the BF/BL region, which is higher than that reported by Brandstrom and Ellegren (2007). International Chicken Polymorphism Map Consortium reported an average mutation density of 5 SNP/kb in the chicken genome (Wong et al., 2004), and that is much lower than what we found in the region, which also indicated high polymorphism of MHC BF/BL region. More than one-third of the variations have a minimum allele frequency above 0.05 in the populations, which meets the requirements of molecular markers for genetic analysis and may be implemented in breeding programs for genetic improvement (Mills et al., 2006, 2011; Vali et al., 2008; Maw et al., 2013).

Among these 18 genes, BF gene, and TAP gene showed high diversity, which is consistent with their antigen presenting roles. The BF genes encode typical MHC class I glycoproteins. The exon 2 and exon 3 of BF genes encoding α1 and α2 domains of MHC-I are related to the resistance of many avian diseases (Hunt and Fulton, 1998; Kaufman et al., 1999; Rogers et al., 2003). TAP molecules are a part of the MHC class I antigen-processing pathway (Pamer and Cresswell, 1998). TAP heterodimer formed by TAP1 and TAP2 transports the antigen peptides into endoplasmic reticulum (ER; Deverson et al., 1990; Trowsdale et al., 1990; Spies et al., 2008). In human, one TAP heterodimer, four tapasin molecules, and four class I-β2m molecules form a peptide-loading complex that assists efficient loading of peptide onto class I molecules (Ortmann et al., 1997). Once the peptide in the lumen of the ER associates with class I molecules, the complex of MHC class I, β2m and the peptide will move to the cell surface, and the peptide will be presented to CD8 + T cells (Pamer and Cresswell, 1998). As key components of the MHC-I antigen presenting pathway, super high polymorphism of BF gene and TAP gene potentially contribute to highly effective antigen presentation and broad-spectrum host resistance to pathogens. Therefore, the high diversity that we found in most of the Chinese local populations implies that they potentially possess a broad-spectrum of disease resistance capability.

Among the domestic populations, NY chicken, GS chicken, XG fowl, and XY chicken were found with the highest polymorphism. These breeds were not yet subjected too strong artificial selection. NY chicken, a local breed in Guangxi province of China, showed the most abundant diversity, but the information about this chicken is limited. Guangxi local poultry resource book described that it was once a pheasant population in Guangxi, China. The habitat of the NY chicken was primarily in the forests, and it was domesticated after capture by the natives. Previous research suggested that RJFs showed extraordinarily high diversity (Hoa et al., 2016), but it was observed with fewer variations in our study. These might result from that only three RJFs were used in this study. High polymorphism of Chinese domestic chickens was also observed by Qu et al. (2006). They found the average heterozygosity of 78 Chinese indigenous chicken breeds was 0.622 by analyzing 27 microsatellite markers. The diversity of WL and CS in our research is lower than that of most Chinese domestic chicken populations, which might be resulted from historical selection for performance characteristics. Izadi et al. (2011) reported that intensively selected commercial populations seem to have low genetic variability in the MHC regions compared with that of the non-commercial flocks, which are less intensively selected. Manjula et al. (2020) reported that compared with commercial breeds, Korea native breeds had high genetic diversity in the MHC-B region, and they showed immune capabilities and genetic potential for resistance to many different pathogens. Hillel et al. (2003) used 22 microsatellite markers to analyze the polymorphisms of 52 chicken breeds from different countries and found that the average heterozygosity of local breeds was 0.50, but that of the commercial chicken breeds was only 0.29, which indicated that long-term intensity selection reduces population diversity. In this study, we found the polymorphisms of line 6_3_ and line 7_2_ were among the lowest, which must be resulted from high-pressure selection and is in good agreement with the other studies (Waters, 1945; Stone, 1975; Bacon et al., 2000).

## Conclusion

We identified abundant genetic variations in the chicken MHC-B-F/B-L region, which not only strikingly expanded the current Ensembl SNP database, but also provided comprehensive data for researchers to further investigate association between variants in MHC and immune traits. The Chinese domestic breeds showed high diversity, which suggest their broad-spectrum resistance to pathogens. Long-term intensity selection significantly reduced diversity of MHC B-F/B-L region of the highly inbred line 6_3_ and 7_2_ chickens.

## Data Availability Statement

All raw sequence data had been deposited in NCBI Sequence Read Achieve (SRA) under the Bioproject number PRJNA728310. The experiment numbers for the 195 chickens are SAMN19075170–SAMN19075364.

## Ethics Statement

The animal study was reviewed and approved by the Ethics Review Committee for Laboratory Animal Welfare and Animal Experiment of China Agricultural University. Written informed consent was obtained from the owners for the participation of their animals in this study.

## Author Contributions

YY performed the experiments, analyzed the data, and wrote the manuscript. HZ supported the samples and provided comments on the manuscript. GY provided comments on the data analysis and revised the manuscript. KW provided comments on the data analysis. ZY, CZ, HY, and JL prepared and detected the samples. NY supported the samples and provided comments on the experiment. LL conceived and designed the experiment, provided comments on the data analysis, manuscript writing, and revision. All authors contributed to the article and approved the submitted version.

## Conflict of Interest

The authors declare that the research was conducted in the absence of any commercial or financial relationships that could be construed as a potential conflict of interest.

## Publisher’s Note

All claims expressed in this article are solely those of the authors and do not necessarily represent those of their affiliated organizations, or those of the publisher, the editors and the reviewers. Any product that may be evaluated in this article, or claim that may be made by its manufacturer, is not guaranteed or endorsed by the publisher.

## Footnotes

1
https://www.ncbi.nlm.nih.gov/

## Supplementary Material

Supplementary Table 1 Primer design for chicken MHC BF/BL region using WaferGen Smartchip Platform.

Supplementary Table 2 The number of SNPs and INDELs in 18 genes.
